# Clinical leadership development: Current practices and future directions for Southern African primary healthcare

**DOI:** 10.4102/safp.v68i2.6294

**Published:** 2026-02-27

**Authors:** Richard Cooke, Angela de Sá, Louis S. Jenkins, Ramprakash Kaswa, Klaus B. von Pressentin

**Affiliations:** 1 Department of Family Medicine and Primary Care, Faculty of Health Sciences, University of the Witwatersrand, Johannesburg, South Africa; 2 Division of Family Medicine, Department of Family, Community and Emergency Care, Faculty of Health Sciences, University of Cape Town, Cape Town, South Africa; 3 Department of Family and Emergency Medicine, Faculty of Medicine and Health Sciences, Stellenbosch University, Cape Town, South Africa; 4 Primary Health Care Directorate, Department of Family, Community and Emergency Care, Faculty of Health Sciences, University of Cape Town, Cape Town, South Africa; 5 Department of Family and Emergency Medicine, George Hospital, Western Cape Department of Health, George, South Africa; 6 Department of Family Medicine and Rural Health, Faculty of Health Sciences, Walter Sisulu University, Mthatha, South Africa

**Keywords:** clinical leadership development, primary healthcare, family medicine, entrustable professional activities, work-based assessment, mentorship and coaching, health system strengthening

## Abstract

Family physicians are uniquely positioned to lead efforts to tackle Southern Africa’s persistent primary healthcare challenges, yet such leadership is often perceived as a top-down process rather than a tangible presence. Drawing on insights from the 2025 South African Academy of Family Physicians (SAAFP) conference workshop, we synthesised leadership behaviours, innovative curricula, and workplace learning approaches, aligning with Entrustable Professional Activities and national unit standards. We outline a 5-year roadmap for clinical trainers, programme leaders, and the SAAFP, including: creating psychologically safe learning environments; embedding 360° feedback; normalising mindfulness and reflective practice; and leveraging mentorship for early-career family physicians.

**Contribution:**

This Open Forum contributes to the *South African Family Practice* journal’s 45-year special collection by reflecting on significant milestones and proposing future directions for clinical leadership and governance. Practical strategies are offered to embed leadership presence, mentorship, and a culture of feedback into training programmes and health system strengthening activities.

## Introduction

### Leadership presence as a catalyst for change in Southern African primary healthcare

Primary healthcare systems in Southern Africa continue to grapple with persistent challenges, including skilled workforce shortages, entrenched hierarchies and inequities exacerbated by social determinants of health. Against a backdrop characterised by volatility, uncertainty, complexity and ambiguity (VUCA) – and increasingly by brittleness, anxiety, non-linearity, and incomprehensibility (BANI) – the role of adaptive, self-aware leadership is more critical than ever.^1,2^ Family physicians, positioned at the nexus of clinical service and community engagement, are uniquely suited to spearhead innovations in clinical governance (CG) and promote health equity, especially as health systems transition towards universal health coverage and the full implementation of the National Health Insurance (NHI) policy.^3^ The recent assent of the NHI by the president in May 2024, highlights the need for inclusive clinical leadership development, emphasising collaboration between public and private sectors. Public and private partnerships, especially at a local district level, may include fostering effective communication, shared care protocols and community engagement to enhance patient transitions, integrated care and equitable access to quality healthcare.^4^

Building on insights from the 2025 National Family Medicine Conference workshop hosted by the South African Academy of Family Physicians (SAAFP) and the growing scholarship on distributed and relational leadership,^5^ this submission proposes practical strategies and a developmental roadmap to strengthen clinical leadership among family medicine registrars and family physicians (FPs) across the region.^6,7,8,9^ By aligning with Entrustable Professional Activity (EPA) 21: ‘Leading a clinical team’ and national unit standards, we advocate for mentorship, experiential learning, and the cultivation of authentic presence as core drivers for transformative leadership development in primary healthcare.^10,11,12,13^ Through this Open Forum, we aim to accelerate the adoption of best practices, foster collaborative communities of learning, equip clinical leaders to navigate complexity and uncertainty, effectively champion equity and meaningfully serve their communities.

### Workshop objectives and process

We synthesised insights from a 90-min interactive workshop at the 27th annual SAAFP national conference held on 06 September 2025 in Midrand, Gauteng, South Africa. The workshop targeted newly qualified and early-career FPs, as well as those involved in workplace-based supervision and mentoring of registrars and early-career FPs. Objectives included exploring links between leadership and CG, developing CG leaders within health systems, and showcasing practical examples such as antimicrobial stewardship rounds, multidisciplinary palliative care rounds, clinical audits, staff management, and the establishment of a safe learning environment.

The workshop process consisted of three phases: (1) scene-setting presentations covering leadership as a way of being, its developmental nature, and the connection between CG and leadership presence, framed by EPA 21 and national unit standards; (2) small-group discussions focusing on self-awareness, additional leadership qualities, barriers and facilitators, and identifying one to two priority steps for clinical trainers and supervisors, academic programmes, and the SAAFP Education and Training Committee; and (3) plenary synthesis to consolidate themes and develop recommendations.

## Current challenges: From process to presence

Clinical governance structures – such as audits, antimicrobial stewardship rounds and pharmaceutical therapeutic committees – are essential but insufficient to drive leadership development on their own. Practice improves most when leaders communicate with clarity, invite diverse perspectives, and foster trust, psychological safety and compassion within their teams. As one presenter at the National Family Medicine Conference observed, ‘Leadership is the presence and soft skills we bring to our roles. It is a way of being rather than simply what we do’.

Experiential entrustment for EPA 21 often plateaus by registrar training in the second or third year, leaving registrars without the necessary exposure to leading meetings, coordinating rosters, managing conflicts and cultivating team culture. Feedback cultures are frequently underdeveloped; teams benefit greatly from scheduled, constructive 360-degree feedback and ‘brave spaces’ for debriefing. A South African study on leadership values found that most participants perceived the healthcare environment as one of relational disconnect and constraining silos and hierarchies. Healthcare professionals found it difficult to share their personal emotions and grapple with sharing their values and feelings in the workplace. This blocking of emotional expression seemed to be institutionalised by the professional training of healthcare practitioners in hierarchies and functional silos.^14^ Finally, public–private collaboration remains inconsistent; leaders must proactively bridge communication gaps now, especially with NHI reforms on the horizon.^15^

Becoming an entrusted leader is a developmental journey marked by psychological growth. Training programmes must incorporate integrated opportunities to build a strong foundation of personal self-awareness, which in turn, supports the cultivation of professional leadership presence and effective practice. Leadership development opportunities within existing training programmes are both feasible and valuable, enhancing the purpose and impact of the CG role that FPs fulfil.

Efforts to transform healthcare must focus on shifting mindsets, embracing diverse perspectives and building teams that trust one another and hold each other accountable. This calls on leaders to question their assumptions, challenge their own views and reflect on the prevailing culture of the health system. Self-awareness and presence allow leaders to navigate this role with greater ease and efficacy. A complex, adaptive health system needs leaders who take a whole-society view of patients and families in communities – seeing communities not as broken or weak, but as whole, with assets that can be utilised. Only when we recognise our communities as true partners can FPs meaningfully empower those they lead and genuinely serve the people in their care; otherwise, we risk disempowering them.

## Insights on current educational approaches and innovations to scale now

### Embed leadership presence within clinical governance

The visible enactment of key microbehaviours needs to be complemented by also fostering a deeper, intentional inquiry into the internal motivations and meta-awareness that underpin them. Micro-level leadership behaviour refers to the observable verbal and non-verbal actions and interactions of leaders with their team members, which have the potential to influence the attitudes and behaviours of those they lead.^16^ While actions such as kindness paired with accountability, inclusive facilitation, routine huddles and debriefings can visibly transform team climate and drive effective CG cycles, their effectiveness ultimately depends on the leader’s heightened self-awareness.^17^ This includes continual reflection on the motivations, beliefs and intentions shaping their responses across different contexts, as well as active attentiveness to how the team perceives their behaviours. Leaders equipped with this meta-awareness are not only able to identify what drives their actions but are also willing and able to adapt their approaches in response to feedback and changing team dynamics, thereby building more authentic, responsive and resilient clinical teams.

A competent clinical leader cultivates insight into the drivers of their decisions and behaviours, regularly reflecting on how these are landing with the team.^18^ Recent literature on leading with kindness unpacks six types of kindness in the clinical context that also have relevance in service and academic relationships, namely: (1) deep listening; (2) evident empathy; (3) generous acts of discretionary effort that go beyond what patients and families expect; (4) timely care that reduces stress and anxiety; (5) gentle honesty in discussions and conversations; and (6) thoughtful support for families and carers.^19^ There is evidence that links kindness to better staff experience and retention, improved teamwork and enhanced patient outcomes.^19^ Crucially, such a leader can adjust their approach when needed – demonstrating psychological flexibility and a deep capacity for self-awareness and other-awareness. This was a focal point in our small-group discussion, where it became clear that describing micro-behaviours alone is insufficient without this internal foundation.

We define effective clinical leadership as the intentional practice of the A-RICH framework, linked to professional behaviour that builds trust by cultivating Agency, Reliability, Integrity, Capability and Humility.^20^ These components may be complemented by relational qualities such as compassion, openness and curiosity (A-RICH+) that underpin a leader’s ability to both understand and shape the micro-behaviours that foster high-functioning, inclusive teams. Compassion involves recognising patients’ suffering and prioritising their well-being. Openness means approaching others without prejudice and valuing diverse perspectives, while curiosity reflects the desire to understand patients’ unique contexts and needs. When leaders are authentic and fully aware of both their influences and the impact of their actions, the desired behaviours and outcomes flow more naturally and sustainably – creating climates of trust, accountability and continuous growth.

### Build mentorship, coaching, and supervisor capability with Next5

District mentorship networks should provide protected time, peer learning and foundational role assessments. The SAAFP Next5 initiative offers a structured approach to support early-career FPs through mentorship and role clarification during their first 5 years.^21^ Beyond traditional mentoring, there is a growing recognition of the value of coaching as a means to develop self-awareness and leadership presence. Coaching, which is less hierarchical and more facilitative, fosters psychological safety, encourages deeper reflection and allows freer exploration of one’s leadership identity. Incorporating coaching approaches, whether one-on-one or group-based, can complement mentoring structures and further empower learners to develop authentic leadership styles.

### Normalise 360-degree feedback and reflective practice in work-based assessment portfolios

Integrate multi-source feedback and entrustment-based discussions into portfolios (EPA 21). Plan feedback sessions deliberately and foster psychological safety. Additionally, as educators, it is imperative for clinical and educational supervisors to critically reflect on and align their own understanding of leadership. Entrusting the development of clinical leaders to faculty presupposes a shared language and collective understanding of what leadership entails. This theme emerged prominently in our workshop planning and group discussions, highlighting the need for ongoing dialogue and self-examination among educators to ensure consistency and clarity in teaching, mentoring and role-modelling leadership presence.

### Create communities of practice and spiral experiential learning

Establishing communities of practice (CoPs) and adopting spiral models of experiential learning create a scaffolded environment for authentic leadership development. Regular opportunities for role-model exposure, learning-by-doing and guided navigation of complex systems are essential, with gradual entrustment aligned to national outcomes.

The experience of revising the Leadership and Governance (L&G) module at the University of Cape Town (UCT) highlights the importance of moving beyond hierarchical structures and recognising that leadership maturity is not synonymous with title or expertise.^8^ Instead, effective leadership – particularly in CG – requires actively cultivating self-awareness and presence. Traditional hierarchies can limit growth, as seen when CG interventions fail to achieve intended outcomes.

The 2022 revision of the UCT module expanded the curriculum from 7–12 sessions, integrating leadership development through group coaching and a five-lens Enneagram profile to deepen self-awareness. Reflective sessions, enhanced by inviting learners from outside the core programme, create greater diversity, enrich communal learning and build leadership capacity. These are paired with practical application: following a guest expert’s theory session (e.g. on running a pharmaceutical and therapeutics committee), participants engage in reflective practice to explore their personal leadership style and impact. This approach disrupts rigid procedural focus and supports discernment, collaboration and authentic team processes.

By integrating new perspectives and creating intentional spaces for self-reflection, the module aims to build a solid foundation of personal leadership. Leadership is nurtured as a presence and ‘way of being’, not merely a set of competencies tied to hierarchy. These CoPs and spiral learning cycles foster environments where leaders continuously revisit and grow into professional roles, anchored by authentic self-awareness. Over time, this empowers clinicians to move from technical expertise towards collaborative, adaptive practice – enabling more effective, equity-focused CG in complex, rapidly changing health systems.

## EPA 21 entrustment thresholds

### What ‘ready’ looks like

While registrars and junior consultants are often adept at the technical and cognitive aspects of CG, authentic leadership is about more than following processes – it is rooted in presence, self-awareness and influence. Leadership presence is revealed in everyday actions: how we communicate, listen, facilitate meetings, write emails or respond in moments of uncertainty. Team members and patients experience it through leaders’ use of body language, tone of voice and empathetic listening during patient care.^22^ It is this presence that draws people in, inspires trust and elevates team performance – much like the unforgettable energy of a compelling speaker who captivates a room.^23^ A workplace culture that emphasises kind leadership is essential for fostering a supportive and effective care environment.^24^

Entrustable Professional Activity 21 articulates a comprehensive approach to leading clinical teams, encompassing not only task delegation and management functions – such as recruitment, line management and performance oversight – but also softer, relational elements.^10^ Key among these is self-awareness: understanding one’s impact, practising kindness and humility, and actively developing reflective habits. Contributing to a positive institutional culture, supporting colleagues and valuing diversity and trust are now recognised as essential, not optional, leadership behaviours.

Crucially, leadership is not confined to formal roles or hierarchical positions. It is a developmental journey, shaped by intentional opportunities for personal growth, feedback and collective learning – whether through spiral learning in training programmes, group coaching or intentional reflection. Both the structure of EPAs and the South African family medicine national unit standards emphasise ethical practice, collaboration and the capacity to care for oneself and others, including managers. As FPs and clinical leaders, making space for self-reflection and personal development is not selfish; it underpins our ability to support and inspire others and to build resilient, effective healthcare teams capable of meeting the complex demands of the district health system.^25,26^

Box 1 illustrates the conceptual connections among the elements of the leadership development loop.

BOX 1 A conceptual flow diagram of the ‘Inclusive Communities of Care Leadership Loop,’ derived from EPA 21, unit standards, and workshop insights into micro-behaviours, feedback culture, and collaboration.

→ **Inputs** (leadership presence and A-RICH+)

- Self-awareness, reflection, humility
- Compassion, openness, curiosity
- Agency, reliability, integrity, capability

→ **Micro-behaviours** (everyday practices)

- Kindness with accountability
- Inclusive facilitation; huddles; debriefs
- Planned 360° feedback; direct public–private communication
- Community feedback loops

→ **Team processes** (EPA 21 tasks)

- Shared leadership; role clarity; performance support
- Roster coordination; conflict resolution; meeting facilitation
- WBA portfolios with entrustment-based discussions

→ **Equity outcomes** (inclusive communities of care)

- Trust and psychological safety
- Continuity across sectors; reduced information gaps
- SDH-responsive partnerships with communities

A-RICH+, agency, reliability, integrity, capability and humility plus compassion, openness and curiosity; SDH, social determinants of health; EPA, entrustable professional activity.

## Recommendations: Directions for the next five years

A pragmatic roadmap links training and service realities. Based on the workshop discussion, we suggest steps to cultivate a leadership culture (see Box 2), tailored to three key stakeholder groups: clinical trainers and supervisors, university programmes and the SAAFP Education and Training Committee.

BOX 2 Steps to create a leadership culture.

- Establish a shared vision of leadership co-created with faculty, registrars and students.
- Role-model collaborative, servant-style leadership in everyday practice.
- Integrate leadership into training (communication, conflict resolution, systems thinking, advocacy).
- Create mentorship and coaching structures; embed Next5 pathways for early-career support.
- Facilitate collaborative decision-making through participatory governance and feedback loops, while acknowledging the positions of other stakeholders to promote and support this shift.
- Promote reflective practice using journaling, 360° feedback, and facilitated discussions. It is essential to define the context carefully, as these processes can be disruptive and challenging for individuals.
- Provide practice opportunities (lead QI projects, chair meetings, coordinate events).
- Celebrate and reward leadership alongside clinical excellence.
- Sustain communities of practice through continuous learning (short courses, fellowships, leadership interest groups).

QI, quality improvement.

### Clinical trainers and supervisors

Collecting feedback from early-career FPs and trainers about their mentorship experiences (and explicitly valuing both formal and informal approaches) can inform improvements and ensure that mentorship structures effectively support leadership development. The following steps were generated from the discussions:

- Schedule regular brave-space debriefs to foster open communication and psychological safety.
- Conduct baseline role assessments to clarify expectations and responsibilities.
- Embed deliberately planned 360-degree feedback cycles to support ongoing professional growth.
- Ensure intentional exposure to EPA 21 (leading a clinical team) in training the second or third year, track engagement and identify opportunities for further development.
- Strengthen supervisor capability through targeted training in human relations, conflict resolution and cultural competence.
- Normalise mindfulness and reflective practice in daily routines.
- Partner with the Next5 initiative to support early-career FPs locally, recognising that while formal mentorship is available, uptake may not be as enthusiastic as anticipated. There is a need to understand better whether mentorship and peer support are occurring more informally and, if so, to adapt pairing and support strategies to meet clinicians where they are.
- Partner with the private sector and various provincial departments of health to align leadership development in clinical service with academic programmes.

### University programmes

The following steps position university programmes to move beyond formal competency checklists, nurturing a new generation of clinical leaders uniquely equipped for the evolving realities of Southern African primary care:

- Adopt spiral leadership curricula that offer repeated, developmentally scaffolded opportunities for leadership practice, ideally incorporating structured group coaching to foster reflection, peer learning and ongoing growth.
- Integrate personality frameworks or lenses (such as the Enneagram) responsibly to promote self-insight among registrars – ensuring these tools enhance, rather than define, individual and team development.
- Expand registrar and faculty portfolios to include multi-source evidence of leadership progress, such as feedback from supervisors, peers and self-reflection, as well as illustrative case experiences and project outcomes.
- Build and sustain multiprofessional communities of practice that connect registrars across faculties, professions and disciplines, offering richer perspectives and modelling collaborative leadership.
- Prepare registrars to navigate workplace uncertainty and constant change, and to engage with EPA 21, as expected from clinical competency committee processes, using practical simulations and real-world scenarios core to the curriculum.

### South African Academy of Family Physicians Education and Training Committee

The SAAFP Education and Training Committee (ETC) can set a clear standard for high-quality, relational, and contextually relevant leadership development for FPs across Southern Africa through these expanded efforts:

- **Intentionally endorse the personal developmental pathways:** Explore what tools and training are available to cultivate self-awareness, self-acceptance, emotion regulation and relational competence that contribute to the psychological maturation of leaders. Facilitation of this personal growth will help ensure that the A-RICH+ qualities and behaviours can more naturally and authentically manifest in future clinical leaders.
- **Develop and publish national guidance for EPA 21:** Provide clear, practical resources outlining micro-behaviours, feedback cultures and creation of brave spaces. This could include best-practice toolkits, sample feedback forms, video guides and implementation checklists to assist clinical trainers and supervisors across all training sites.
- **Endorse and standardise A-RICH+ relational qualities:** Explicitly incorporate relational qualities (openness, curiosity, compassion) into the expanded A-RICH framework within assessment rubrics and curriculum content. This highlights the importance of fostering authentic and relational leadership inspired by ubuntu. Include these qualities directly in EPA assessment rubrics, faculty development sessions and registrar induction materials to promote a value-based approach to leadership.
- **Institutionalise Next5 mentorship within district activities:** Move beyond ad hoc mentorship by formalising regular mentor-mentee pairings, protected time for mentorship conversations and periodic group or peer meetings. Establish clear outcome metrics for evaluating the reach and impact of mentorship, ensuring consistent support for early-career FPs and a seamless transition from training to clinical practice.
- **Monitor and evaluate implementation:** Collect regular feedback from trainers, registrars and stakeholders on the effectiveness and uptake of these initiatives. Use surveys, workshops and programmatic data to identify gaps and areas for ongoing improvement, ensuring that guidance remains relevant and practice-focused.
- **Facilitate continuous faculty and leadership development:** Offer short courses, webinars and leadership interest groups to help faculty and supervisors stay up-to-date with emerging best practices, maintain engagement and build a cohesive leadership pipeline across the profession. These activities could complement current efforts, including the 5-day Training of Clinical Trainers course and the accreditation of clinical trainers.

## Conclusion

As the *South African Family Practice* journal celebrates 45 years of advancing family medicine, this Open Forum highlights the important truth that leadership is about presence, not just position. Our workshop revealed that authentic clinical leadership stems from self-awareness, kindness and everyday behaviours that foster trust and psychological safety. We can transform CG from a procedural duty into a catalyst for equity and resilience by embedding these principles into EPA 21, mentorship networks like Next5 and spiral learning curricula.

In the context of this Open Forum, workshop participants humbly recognised that leadership behaviours were the main focus of the discussion. Although the importance of FPs gaining insight and self-awareness to become effective leaders was acknowledged, it was only briefly mentioned, suggesting that further efforts are needed. Importantly, we must ensure that, as we promote the outcomes of mature clinical leadership, we do not set the cart before the horse by neglecting the foundational developmental processes linked to psychological maturation such as self-insight, self-regulation, and unconditional self-acceptance that are prerequisites for authentic engagement with others and for the genuine emergence of the A-RICH+ qualities.

The next five years offer a crucial opportunity to create inclusive care communities, normalise feedback cultures and invest in faculty development that embodies collaborative leadership. These steps align with the SAAFP and its journal’s legacy of promoting responsive, patient-centred systems, as well as their commitment to enhancing primary healthcare in Southern Africa. By embracing leadership as a way of being, FPs can navigate complexity and champion health equity, ensuring that CG reflects the values of trust, compassion and shared purpose.
